# Flexible Printed Monolithic-Structured Solid-State Dye Sensitized Solar Cells on Woven Glass Fibre Textile for Wearable Energy Harvesting Applications

**DOI:** 10.1038/s41598-018-37590-8

**Published:** 2019-02-04

**Authors:** Jingqi Liu, Yi Li, Sheng Yong, Sasikumar Arumugam, Steve Beeby

**Affiliations:** Smart Electronic Materials and Systems Group, School of Electronic and Computer Science, University of Southampton, Highfield, Southampton, SO17 1BJ UK

## Abstract

Previously, textile dye sensitised solar cells (DSSCs) woven using photovoltaic (PV) yarns have been demonstrated but there are challenges in their implementation arising from the mechanical forces in the weaving process, evaporation of the liquid electrolyte and partially shaded cells area, which all reduce the performance of the cell. To overcome these problems, this paper proposes a novel fabrication process for a monolithic-structured solid-state dye sensitized solar cell (ssDSSC) on textile using all solution based processes. A glass fibre textile substrate was used as the target substrate for the printed ssDSSC that contain multiple layers of electrodes and active materials. The printed ssDSSC on textile have been successfully demonstrated and compared with a reference device made with the same processes on a glass substrate. All PV textile devices were characterized under simulated AM 1.5 conditions and a peak efficiency of 0.4% was achieved. This approach is potentially suitable for the low cost integration of PV devices onto high temperature textiles, but to widen the range of applications future research is required to reduce the processing temperature to enable the device to be fabricated on the standard fabric substrates.

## Introduction

Electronic textile (e-textile) based wearable technology has been demonstrated for various applications in recent years, for example, intelligent biomedical garments for monitoring, diagnosing and treatment of medical conditions^1,2^, wireless cardiac signal monitoring in sports^2,3^ and military clothing integrating fabric antennas to support networks and communications^4,5^. However, in every e-textile application the issue of supplying power remains a significant challenge. E-textiles are typically battery powered using conventional rigid batteries that alter the characteristics (e.g. feel and drape) of the fabric. Batteries also require periodic replacement and are therefore inconvenient to use and dispose of. One alternative approach to batteries is to investigate energy harvesting technologies on textiles. Energy harvesting involves the conversion of ambient energy (e.g. kinetic, thermal or light) into electrical energy and this can provide a long-term, easy to use power supply that can potentially be embedded within the textile (i.e. the fabric is functionalized such that it becomes the energy harvester)^6^ to meet the individual needs. Harvesting light energy through textile based solar cells are expected to be a promising approach. Functionalizing textiles is not straightforward since they are complex structures formed from a wide variety of fibres using many different manufacturing techniques. Fabrics also place constraints on the technologies that can be used (e.g. limiting process temperatures) and the surface roughness presents a challenging substrate on which functional film must be deposited. There are many existing examples of solar cells on fabrics that use conventional rigid silicon (glass) or plastic solar cells, as standalone PV devices, which are attached onto the fabric as a functional patch^7^. This approach alters the feel of the textile dramatically making the fabric relatively inflexible and non-breathable, and the fabric itself has no added functionality.

This paper presents an investigation into the fabrication of solid-state dye sensitized solar cells directly (ssDSSC) on fabric substrates. The constraints of the fabric substrate mean that the existing processes and technologies cannot be simply applied directly onto the textile. In recent years, organic, dye-sensitized, and perovskite solar cells have demonstrated great potential within the photovoltaic market^8^. The dye sensitized solar cell (DSSC), introduced by O’Regan and Gratzel^9^, is a promising candidate for flexible plastic and e-textile applications because of it’s the straightforward and low cost fabrication processes required and high energy conversion efficiency^10–12^. The original DSSC consisted of two rigid glass substrates incorporating the electrodes with a liquid electrolyte injected between them. Yin *et al*. fabricated plastic DSSCs using flexible Indium tin oxide/polyethylene naphthalene (ITO/PEN) substrates as front and back electrodes with a liquid electrolyte in-between the sealed plastic substrates^13^. This device achieved a power conversion efficiency (PCE) of 5.8%. Opwis *et al*. fabricated DSSCs on a glass fibre textile which had a screen printed polyamide film to smooth out the textile and a sputtered titanium bottom conductive layer. The plastic ITO/PET substrate was used as the top electrode with the liquid electrolyte injected in-between the sealed textile photo anode and the ITO plastic top electrode^14^. The devices reached a PCE of 1.1%.

Another approach to realise DSSC textiles is to functionalize the yarns^15–17^ and fibers^18–20^, which can be woven in to a textile. Very recently, Zhang *et al*. reported a DSSC textiles fabricated using Polybutylene terephthalate (PBT) polymer yarns woven into different fabric structures. This used a liquid electrolyte^21^ and achieved a PCE of 1.3% for a single fibre, which is the highest reported to date for fibre based DSSCs. This approach presents a challenge in large area applications to connect a number of crossed, cylindrical yarn solar cells that have been woven into the textiles^22^. Furthermore, the limitation of the bending radius curvature^23–25^, as well as the fibres are tending to break very easily which will lead degrade^26^ in cell performance. In addition, when woven into textiles, they are partially shaded which may reduce the power conversion efficiency. All of these photovoltaic yarns use conventional liquid electrolyte DSSCs, which suffer from leakage, corrosion and long-term stability problems in practical applications^25^.

These issues can be avoided using hole transport materials or solid electrolyte. Since first reported by Bach *et al*. in 1998^27^, the amorphous organic material Spiro-OMeTAD (*N*^2^,*N*^2^,*N*^*2′*^,*N*^*2′*^,*N*^7^,*N*^7^,*N*^*7′*^,*N*^*7′*^-octakis(4-methoxyphenyl)-9,9^*′*^-spirobi[fluorene]-2,2^*′*^,7,7^*′*^-tetraamine) has been considered as the most promising solid electrolyte for ssDSSCs^28,29^. Hardin *et al*. fabricated ssDSSC on fluorine doped tin oxide (FTO) glass substrates. In this work, the TiO_2_ compact layer (CL) was deposited using spray pyrolysis followed by doctor blading a TiO_2_ nanoporous layer for dye sensitisation and then spin coating the solid electrolyte spiro-OMeTAD layer^30^. The devices demonstrated a PCE of 2.7% and 2.8% for the evaporated silver (Ag) and laminated silver nanowire (AgNW) top electrodes respectively. Recently, Margulis *et al*. reported the same architecture of ssDSSCs on FTO glass substrates but after spin coating the solid electrolyte, a PEDOT: PSS layer was spin coated to provide a better contact with the spray coated AgNW top electrode. In this case, the device demonstrated an improved PCE of 3.7%^29^.

Printing techniques are an established processing technique for realising e-textiles^31^ and is a straightforward technique for adding functionality on top of any conventional (non electronic) fabric. The device presented in this research paper is fabricated using a novel combination of solution based deposition techniques including screen printing, drop casting and spray coating. All three deposition techniques are compatible with textile manufacturing. Screen printing, for example, is a common approach for patterning textiles with colour inks bonding to yarn fibre and can withstand washing and friction. Drop casting can be performed using dispensing equipment to deposit an isolated droplet or continuous liquid film and this has been fully automated in inkjet printing^32^. Spray coating is a blanket coating process used in the textile industry to deposit continuous films across large fabric areas. For example, it has been used to back coat upholstery fabrics with hot melt polymer. With these processes there is an ongoing research challenge to achieving the uniformity and consistency required for function electronic devices across a large surface area on different textile surfaces. This work has investigated the use of spray coating through a shadow mask to achieve patterned functional thin films and the combination of processes demonstrates the promising potential of solution based processes for that large scale manufacture of e-textiles devices and functional layers on the surface of a fabric.

Previously, we have investigated liquid state DSSC on Kapton and polyurethane coated polyester cotton fabric that demonstrated an efficiency of 7.03% and 2.78% respectively^33^. This device structure involved the fabrication of the photo anode on Kapton and fabric substrates, but rigid FTO glass was used as the top electrode. As a result, the DSSC remained inflexible and the liquid electrolyte is unsuitable for fabricating textile devices because it leaks and evaporates through the fabric. We have also previously investigated the properties of the TiO_2_ layer with processing temperature. DSSC were fabricated on FTO glass and demonstrated an efficiency of 7.41% but it was found the TiO_2_ layer requires a minimum processing temperature of 450 °C to sinter the film and achieve the this efficiency^34^.

Therefore, to achieve a flexible and stable DSSC on textile, this paper presents details of an investigation into the fabrication of a two-dimensional ssDSSC fabricated on woven high temperature glass fibre textile substrates. Textile ssDSSC were printed directly on uncoated glass fibre substrates and on glass fibre textiles planarised using a liquid polyimide film. All materials used can withstand the required 450 °C processing temperature and the performance in each case is compared with a reference structure fabricated on an FTO glass substrate. Devices have been characterised using X-ray diffraction (XRD), PCE and transmittance measurements, atomic force microscopy (AFM) and electrochemical impedance spectroscopy (EIS) has also been used to characterise the electrochemical processes occurring within the glass and fabric ssDSSCs.

## Results and Discussion

**Figure 1:**
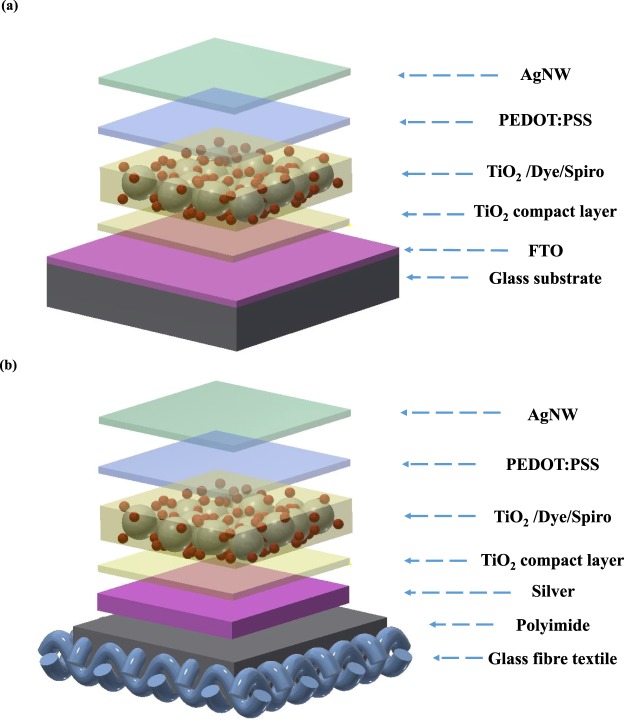
Isometric view of the schematic diagram, (**a**) FTO glass based ssDSSC device structure; (**b**) textile based ssDSSC device structure.

**Figure 2:**
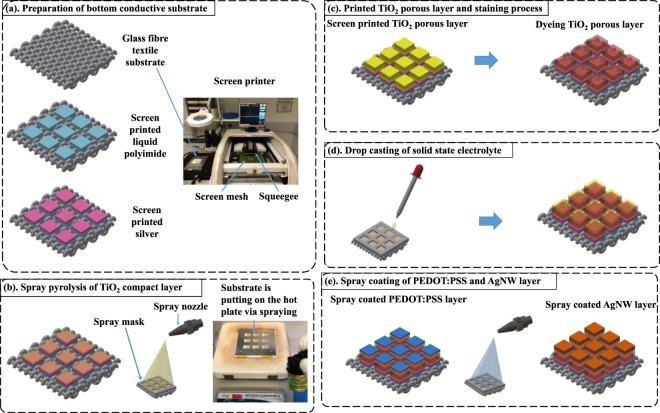
Fabrication process diagram of textile based ssDSSC, (**a**) screen printing polyimide and silver layer to form the bottom conductive substrate, (**b**) spray pyrolysis of TiO_2_ compact layer, (**c**) screen printed TiO_2_ porous layer and staining process, (**d**) drop casting of solid state electrolyte, (3) spray coating of PEDO:PSS and AgNW layer.

The textile based ssDSSC is a flexible 2D planar structure that is a variation of the version fabricated on the glass substrate as shown in Fig. 1(a,b). All functional layers on both glass and textiles are same, except for the bottom electrode, which is the FTO on glass and a thick film silver layer on the textile substrates. The fabrication process for monolithic-structured ssDSSC on a single textile consists of five main steps as shown in Fig. 2(a–e), where all functional layers were directly deposited onto the textile substrates using processes such as screen printing and spray coating. To fabricate the ssDSSC, after initial investigations on uncoated textiles, a flexible polyimide layer was required to reduce the surface roughness of fabric. The use of the liquid polyimide to planarise the surface of the fabric is different to the polyurethane based material typically used due to the temperature limitation of the polyurethane film^35^. Next, the silver bottom electrode was screen printed onto the dried polyimide and this serves to further reduce the surface roughness of the textile substrate as shown in Fig. 2(a). The screen design ensures that the functional layers are only printed where required, and the effect on the fabric properties, such as flexibility and breathability, is minimized.

Figure 2(b) shows the spray pyrolysis deposition of TiO_2_ CL using a pre-designed shadow mask aligned with the silver bottom electrode on the fabric substrate. There are many parameters that influence the uniformity, roughness and coverage of the TiO_2_ film on the substrates during spraying. This includes the flow rate of the functional ink concentration, the distance between the spray nozzle and the substrate and the gas flow pressure that carries the droplets to the substrates. A spray pressure of 0.3 bar was used for all the functional layers and the spray distance for the CL film was 12 cm. The TiO_2_ CL was used to avoid direct contact of the solid electrolyte to the conductive bottom electrode that would otherwise short circuit the cell. The mesoporous TiO_2_ layer was deposited on the TiO_2_ CL and sintered to form photo anodes and this was followed by the dye staining process depicted in Fig. 2(c). Figure 2(d) shows the deposition of freshly prepared solid state electrolyte solution, which was drop cast onto the dye stained devices and left for 16 hours to allow the solid state electrolyte to fully infiltrate into the TiO_2_ mesoporous film. To complete the fabrication, the devices are spray coated with PEDOT: PSS and AgNW top electrodes as shown in Fig. 2(e).

### Results and discussion for ssDSSCs on FTO glass

**Figure 3:**
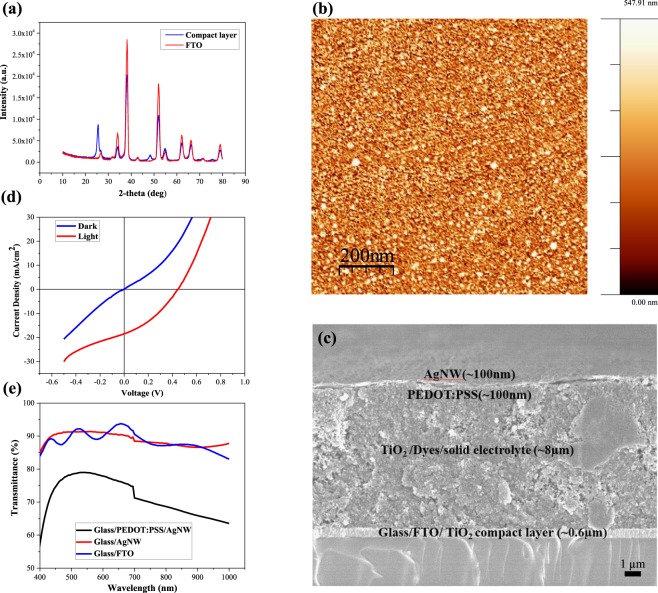
(**a**) XRD spectra of the CL TiO_2_ film prepared by spray pyrolysis on FTO glass substrate, (**b**) AFM graph for TiO_2_ compact layer (CL) on FTO glass substrates, (**c**) FESEM for the cross sectional solid state DSSC device on FTO glass substrates, (**d**) J/V graph for the FTO glass substrates, and (**e**) transmission graph for the AgNW and PEDOT:PSS/AgNW glass substrates.

Figure 3(a) shows XRD results for the TiO_2_ CL compared with FTO reference glass substrates. For XRD, the films were illuminated at a constant incidence angle of 0.2° with a wavelength of 1.54 Å (Cu Anode) and incident energy of 40 keV. The XRD patterns of FTO/glass with the CL TiO_2_ layer, shows a strong diffraction peak of TiO_2_ at 25.5°, which is due to the thin thickness of TiO_2_ layer. The CL TiO_2_ sharp peak is attributed to the existence of a crystalline surface compared to the FTO reference glass substrates. The similar XRD pattern for the CL TiO_2_ and FTO was reported by Charbonneau *et al*.^36^. The crystalline properties of the TiO_2_ CL was further confirmed by observing the morphology of the CL film using an AFM as shown in Fig. 3(b). The surface of the film shows a granular domain interface with an RMS surface roughness value of 131 nm. The CL is well densified with the small crystals formed as desired each estimated to be a few tens of nanometres in length. Moreover, the 1 × 1 µm region scanned in the AFM appears to be free of pinholes despite its low thickness.

To measure the thickness of each functional layer, a cross-sectional image of the ssDSSC was obtained using a SEM as shown in Fig. 3(c). The CL TiO_2_ is not clearly visible as it is only 100–200 nm thick. A 8 µm thick layer of TiO_2_ paste was screen printed directly onto the CL, which was previously identified as the ideal thickness^9^. As can be seen in Fig. 3(c), the solid electrolyte has completely impregnated the dye sensitised TiO_2_ film and hasn’t left a residual solid electrolyte layer that could cause current leakage or block the incident light. PEDOT: PSS and AgNW where then spray coated with a thickness of 100 nm. Table 1 summarises the measured and characterised results of the both FTO glass based and textile based devices. Figure 3(d) shows the J/V curve for the FTO glass based device. The device demonstrated an efficiency of 2.8% with an open circuit voltage (V_OC_) of 0.44 V, fill factor (FF) of 0.34 and current density (J_SC_) of 18.5 mA/cm^2^. The J/V curve shows a decrease in series resistance that suggests there is no barrier to the charge transport between the optimized functional layers. The transmittance spectra plot of PEDOT: PSS/AgNW/glass substrate is shown in Fig. 3(e) alongside a standard FTO electrode on a glass substrate for reference. Topside illumination is used through the PEDOT: PSS/AgNW layers and therefore it is should be as transparent as possible. The PEDOT: PSS/AgNW coated electrode shows high transmittance characteristics of 70–80% in the visible region of 450–850 nm.

**Table 1:** Photovoltaic measurements of the FTO glass and glass fibre textile ssDSSC devices.

Device	V_OC_ (V)	FF	J_SC_ (mA/cm^2^)	PCE (%)
FTO glass	0.44	0.34	18.59	2.83
Bare Textile	0.22	0.23	8.26 × 10^−5^	4.14 × 10^−6^
Liquid polyimide coated glass	0.31	0.25	5.2	0.4

### Results and discussion for textile ssDSSCs

**Figure 4:**
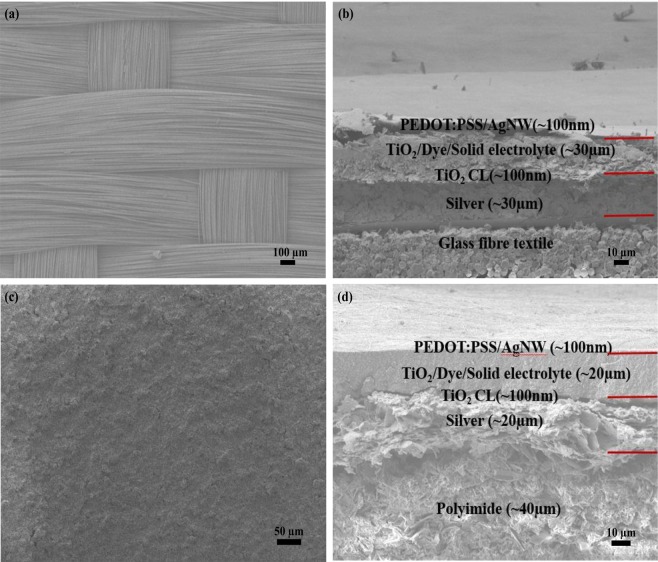
FESEM images of (**a**) Plan view of bare glass fibre fabric and (**b**) Cross sectional view of the printed bare textile ssDSSCs, (**c**) liquid polyimide surface on the glass fibre woven textiles, (**d**) Cross sectional view of the printed liquid polyimide coated textile ssDSSCs.

With the optimisation of the fabrication conditions on the rigid glass substrate, glass fibre textile ssDSSC devices were fabricated with the same steps. Figure 4(a) shows the SEM micrograph of glass fibre textile used in this research. This was initially planarised and made conductive by screen printing a silver layer with a thickness of 30 µm. The surface roughness (R_a_) values of glass fibre textile before and after printing the silver layer is 70.5 µm and 26.1 µm respectively. Whilst it shows the surface roughness of the textile was reduced by the silver, it is still far from ideal for the subsequent deposition of reliable functional layers. The nanoporous TiO_2_ paste was screen printed three times to achieve a smooth continuous layer with a final thickness of around 30 µm as shown in Fig. 4(b). This is substantially greater than the previously identified target thickness of 8 μm but is unavoidable due to the rough textile surface.

**Figure 5:**
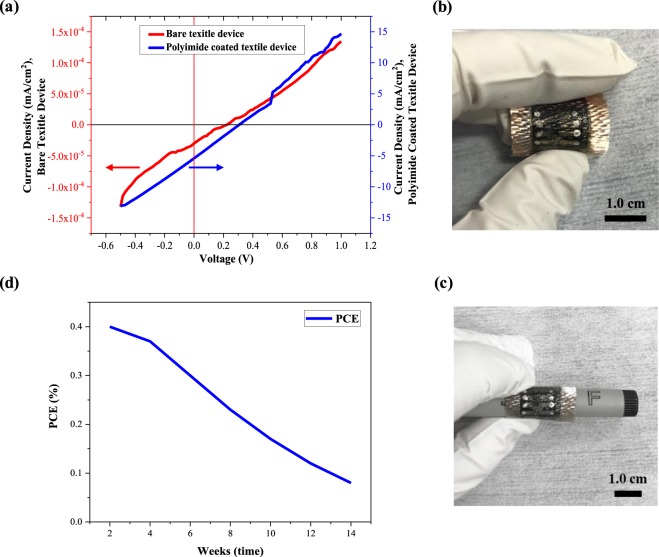
Photograph of (**a**) J/V curve of ssDSSCs fabricated on bare glass fibre textiles and polyimide coated glass, (**b**) Polyimide coated glass fibre textile device under bending, and (**c**) device rolling on a pen, (**d**) the PCE versus time regarding stability in air for polyimide coated glass fibre textile.

The initial glass fibre ssDSSCs with the 30 µm thick TiO_2_ layer demonstrated an efficiency of 4.14 × 10^−6^% with a V_OC_ of 0.22 V, FF of 0.22 and J_SC_ of 3.1 × 10^−5^ mA/cm^2^. The increased thickness of the TiO_2_ means the glass fibre ssDSSC is much less efficient than the FTO glass version with the 8 µm thick TiO_2_ (Table 1) which is ultimately due to the surface roughness of the textiles. Figure 5(a) shows the J/V curves of the fabric ssDSSC that indicate there is resistance to charge movement between the functional layers due to the excessive and uneven film thicknesses. The fabricated ssDSSCs on woven textiles can be seen in Fig. 5(b).

It was therefore necessary to further reduce the surface roughness of the glass fibre textile substrates using a screen printed liquid polyimide layer. The field emission scanning electron microscope (FESEM) image of the top surface of the liquid polyimide surface is shown in Fig. 4(c) and the measured surface roughness (R_a_) of the polyimide layer was 10 µm. As can be seen from Fig. 4(d), a 40 µm thick liquid polyimide layer was printed and the textile appears flat compared to the bare textile surface. The surface roughness was further reduced to 7 µm by the screen printed 20 µm conductive silver bottom electrode. This smoother surface enabled the thickness of the TiO_2_ layer to be reduced to 20 µm, which reduced the resistance in the functional layer. Following the deposition of all functional layers, the device showed a maximum PCE of 0.4% with a V_OC_ of 0.31 V, while the FF and J_SC_ were 0.25 and 5.2 mA/cm^2^ respectively. The PCE of the textile devices improved significantly because of the smooth flat surface that enabled the even deposition of the subsequent functional layers. This is the first reported fabrication of ssDSSCs on textiles with a PCE of 0.4%. The device also demonstrated good stability with a negligible reduction in PCE after it was kept in air for a period of two weeks without encapsulation. In total, five cells obtained 0.4% with the remaining cells obtaining PCE values in the range of 0.1–0.4%. Devices failures were due to short circuits from either the top and bottom electrode contacting or peeling away from the functional layers around the edges.

The PCE values achieved for the textile ssDSSCs are, however, not comparable to the PCE values achieved on the rigid glass slides. There are two factors reducing the textile ssDSSC’s PCE values. Firstly, the sheet resistance of the printed silver bottom electrode was 22 kΩ/□ whereas the FTO coated glass substrates was 10 Ω/□. This corresponds to an increase in the series resistance of the textile cells resulting in lower current density and PCE. This could be improved by depositing binder free conductive layer using, for example, a silver precursor. Secondly, despite reducing the surface roughness of the printed silver electrode on the polyimide coated textile, it is still two orders of magnitude greater than the FTO coated glass slide. This increases the surface roughness of the subsequently deposited functional layers and the Ra value is greater than the optimised TiO_2_ film thickness of 8 μm identified on the glass substrate. A TiO_2_ film thickness of 20 μm is required o the textile to achieve a continuous film and the increased thickness reduces the PCE due to recombination. Further reductions in textile surface roughness are required to reduce TiO_2_ thickness by, for example, increasing the polyimide coating thickness but this would reduce flexibility, as discussed below.

The flexibility of the cells has been defined by determining the effect of repeated bending deformation on device PCE. Figure 5(c) shows the fabricated device under bending around a pen with a radius of 0.5 cm. Actual cyclical bending tests were performed using an automated bending test rig around and devices where repeatedly bent around a radius of 2.5 cm and devices were first encapsulated to prevent abrasive wear during the test. The encapsulated cells were tested with the top AgNW electrode side facing up placing the active films in tension. After 10 bending cycles there was a 60% reduction in efficiency, and after 30 cycles the devices failed. The strain induced in the cell during bending caused the functional layers to mechanically fail due to the polyimide layer cracking. The amount of strain transferred to the device during bending is a function of the thickness and stiffness of the substrate and the polyimide coated glass fibre textile is quite stiff compared with a conventional textile. The strain in the polyimide and device layers can be alleviated by increasing the thickness of the encapsulation layer such that the cell is positioned closer to the neutral axis reducing the strain experienced during bending^37^. Regarding stability in air, the PCE versus time is shown in Fig. 5(d). The encapsulated cells showed no reduction in PCE after 15 days, and after a further 30 days storage the PCE had reduced by around 0.03%. However, after 100 days the PCE had reduced by 80%. This indicates that the encapsulation layer initially protects the devices against exposure to air but this protection fails over time due to the permeability of the encapsulation layer.

**Figure 6:**
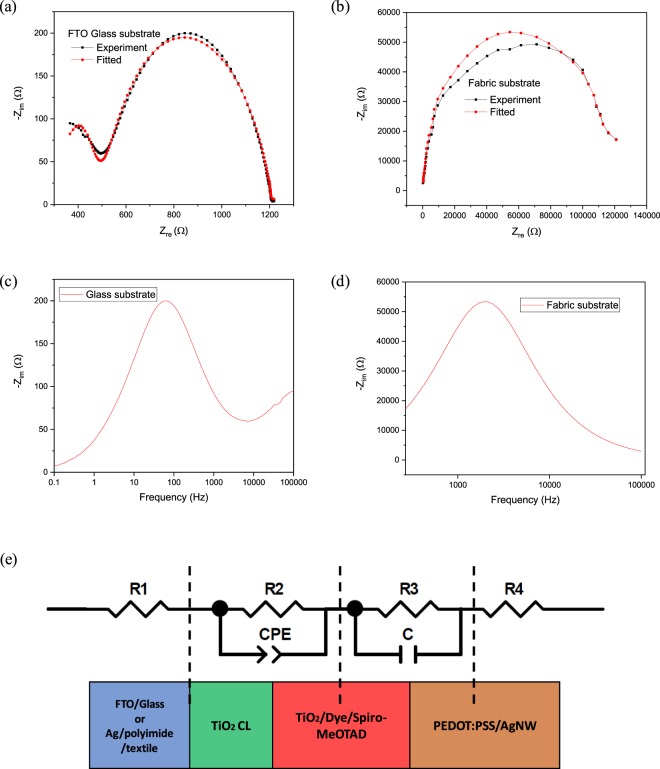
(**a**) Nyquist plot of ssDSSCs on FTO coated glass substrates. (**b**) Nyquist plot of textile ssDSSCs with screen printed polyimide interface layer. (**c**) Bode plot of ssDSSCs on FTO coated glass slide. (**d**) Bode plot of textile ssDSSCs with screen printed polyimide interface layer. (**e**) Schematic diagram of ssDSSCs structure that can be simulated using resistor (R), capacitor (C) and constant phase element (CPE) for glass and fabric (ignoring R3) based device.

In a solar cell, electron transport, recombination and lifetime directly influence the photovoltaic performance, and these are affected by device impedance, which can be studied using electrochemical impedance spectroscopy (EIS). EIS was performed on both the glass and fabric based ssDSSCs, which were measured at a 10 mV forward bias under dark conditions and the Nyquist and bode plots are shown in Fig. 6(a–d). The equivalent circuit model for the glass substrates used to fit the experimental curve is shown in Fig. 6(e) and the fitted data results are given in Table 2. Figure 6(a) shows the Nyquist plot for the glass based device. There is a small semicircle in the high frequency range (Z_re_ 300 to 500 Ω) that is correlated to the active layer/PEDOT: PSS and AgNW electrode interface (R3 in the equivalent circuit). The large semicircle is in the low-frequency range (Z_re_ 500 to 1200 Ω), and is associated with the active layer:TiO_2_ CL interface (R2 in the equivalent circuit) and the constant phase element (CPE) is the capacitance of this interface. The larger semicircle is pre-dominantly associated with charge transfer from the dye to the porous TiO_2_ layer. The values of R2 and R3 directly indicate the electrochemical activity across the junction of the cells and the lower these are the better the device performance. As shown in Table 2, R2 and R3 for the glass ssDSSC (PCE of 2.83%) are 0.72 and 165 kΩ respectively. Figure 6(b) shows the Nyquist plot of the fabric ssDSSC. The shape of this plot is different to the glass ssDSSC and there is only a single large semicircle (Z_re_ 1000 Ω to 120 kΩ). The absence of the smaller semicircle in the high frequency range is due to the challenge of probing the soft fabric. The glass device was tested using crocodile clips device but these pinched through the functional layers and caused the fabric ssDSSC to fail. The fabric DSSC was therefore tested using a 0.2 mm area probe but this was found to penetrate the 100 nm thick top electrode and contact the active layer directly. Hence resistor R3 can be ignored in the circuit model for the fabric ssDSSC curve as shown in Fig. 6(b). Table 2 shows an increased R2 value of 120 kΩ, which indicates a higher rate of recombination due to the increased thickness of the TiO_2_ layer for the fabric ssDSSCs.

**Table 2:** Parameters obtained from the electric impedance spectra (EIS) of the solid-state DSSCs based on FTO coated glass slides and screen printed polyimide glass fibre textile. (R equals R1 plus R4.).

Device	R* (kΩ/cm^2^)	R2 (kΩ/cm^2^)	R3 (Ω/cm^2^)	CPE (F)	Bode peak (Hz)	Electron lifetime *T*_n_ (ms)
FTO glass	0.36	0.72	165	3.3 × 10^−5^	66.02	2.41
Liquid polyimide coated glass	0.54	120	—	1.34 × 10^−9^	2034	7.82 × 10^−2^

*R = R_1_ + R_4_.

To support the Nyquist analysis, bode plots were used to investigate the lifetime of the electron transfer in the ssDSSC photoanode and solid state electrolyte interface. From the bode plot, the electron lifetime (τ_n_) could be obtained from the peak angular frequency using the following equation.

$${\tau }_{n}=\frac{1}{2\pi {f}_{max}}$$

Where f_max_ is the maximum peak frequency. The Bode plots shown in Fig. 6(c,d) indicate that maximum peak frequencies of 66 Hz and 2034 Hz and the corresponding electron lifetime is 2.4 and 7.82 × 10^−2^ ms for the glass and textile based ssDSSCs respectively. The longer the electron lifetime leads to an increased chance of recombination in the fabric ssDSSC and this again reflects the thicker active layer.

## Conclusions

A fully printed textile functioning ssDSSC on a woven glass fibre textile has been demonstrated and this work has identified the influence of the surface roughness of the woven glass fibre textiles. The bare glass fibre textile required a TiO_2_ thickness of 30 μm leading to an efficiency of 4.14 × 10^−6^%. The textile surface was planarised using a liquid polyimide and this enabled a TiO_2_ thickness of 20 μm resulting in an efficiency of 0.4%. The optimised TiO_2_ thickness on the glass ssDSSC was 8 μm but to achieve this a thicker layer of polyimide would be required. However, whilst the polyimide can withstand the processing temperatures used throughout this work and it is flexible to a degree, it does still increase the stiffness of the fabric and cracks after cyclical bending causing devices to fail. Further work is required to improve the flexibility of the polyimide without compromising its ability to survive at high temperature. The cell encapsulation process greatly improves the ability of the device to avoid the effects of oxidation and, by optimizing the encapsulation thickness, it could be used to reduce the strain in the device and polyimide layers thereby improving flexibility. This work has demonstrated the potential for fabricating ssDSSCs directly on fabric using solution based processes suitable for continuous roll-to-roll manufacturing. This PV device configuration could also be fabricated on standard textiles such as cotton, but the high temperature required by the TiO_2_ CL is an issue and further research work is required to reduce the processing temperature required by the compact layers to less than 150 °C. EIS analysis indicated that glass based device showed a higher charge transfer and lower recombination rate compared to the fabric ssDSSC. This reinforces the importance of reducing the thickness of the TiO_2_ layer in order to reduce the resistance in the fabric ssDSSC to obtain a higher efficiency.

## Methods

### Materials

FTO coated glass substrates with the thickness of 1.0 mm and a resistivity of 10 ohm/sq were purchased from Solaronix (TCO10-10). The plain woven composite glass fibre textile was supplied by Valmiera Glass. The screen printed liquid polyimide, was purchased from Epoxy (TV-1003). The screen printable silver paste was purchased from Dupont (Dupont 5000). The compact layer solution of titanium di-isopropoxide bis(acetylacetonate 75% of isoproponal) was obtained from Sigma-Aldrich (325252). The TiO2 paste is a mixture of rutile and anatase, (Sigma-Aldrich 700355) with an average pore size of around 15 nm to 20 nm. The ruthenizer 535-bisTBA dye sensitizer was obtained from Solaronix (ref. 21623). The ingredients for the solid-state electrolyte were: bis(trifluoromethane)sulfonimide lithium salt (544094), Cholorobenzene(284513), tert-butylpyridine (142379), acetonitrile (271004) and N^2^,N^2^,N^2*′*^,N^2*′*^,N^7^,N^7^,N^7*′*^,N^7*′*^-octakis(4-methoxyphenyl)-9,9^*′*^-spirobi[fluorene]-2,2^*′*^,7,7^*′*^-tetraamine (spiro-OMeTAD) (792071), all purchased from Sigma-Aldrich. CleviosTM PEDOT:PSS was supplied by Heraeus and the AgNW solution was purchased from Nanopyxis Ltd.

### Experimental procedures

An automated DEK 248 screen printer was used in all printing steps in this work using pre-designed polyester 75/55 mesh with a 45 µm diameter thread and an emulsion thickness of 5 µm. The glass fibre textile was cut into 15 cm × 15 cm, attached to an alumina tile by a Kapton tape, and placed on the substrate stage holder of the screen printer prior to printing. A squeegee speed 70 cm/min was used throughout. The printing gap for the liquid polyimide layer was set to 1.2 µm for the first two deposits and increased to 1.4 µm for the last two deposits. The screen printed polyimide layer was subsequently annealed at 150 °C and 275 °C for 45 minutes respectively in an oven. For the silver bottom conductive layer, the printing gap was set to 1.6 µm for the first deposit and increased to 1.8 µm for the second deposit. The silver film was cured at 150 °C for 30 minutes.

A thin TiO_2_ CL was deposited via spray pyrolysis using air as the carrier gas. A solution of titanium di-isopropoxide bis(acetylacetonate) was sprayed onto the silver coated glass fibre textile substrate which was pre-heated on a hot plate at 150 °C. After printing the TiO_2_ CL, the textiles substrates were annealed at 500 °C for half an hour. The mesoporous TiO_2_ films were screen printed with a printing gap of 1.8 µm using the commercially available paste and slowly heated to 500 °C for 30 min in an oven in an oxygen environment. The film was cured at 450 °C for 30 minutes, after which it was immersed into a dye solution for 16 hours for staining. The dye solution was Ruthernizer 535-bisTBA in ethyl alcohol solution (100 mg dye powder added to 15 ml of ethyl alcohol). The dye-loaded devices were carefully washed with ethanol to remove loosely bound dye molecules and minimise the resistance of the films. Then the devices were transferred to a nitrogen filled glovebox for drop casting 3 µl of the solid electrolyte onto the surface of the dye loaded TiO_2_ layer. The organic hole conductor, or solid electrolyte solution, was made by dissolving 260 mg of spiro-OMeTAD in 1.2 ml of chlorobenzene and then adding 25 μl of tert-butylpyridine and 40 μl of bis(trifluoromethane)sulfonamide lithium salt solution. The lithium salt contains 170 mg lithium salt and 1 ml of acetonitrile. The doped spiro-OMeTAD solution was allowed to infiltrate the TiO_2_ for 12 hours in a nitrogen filled glove box. Then a thin layer of PEDOT:PSS was spray coated inside the glove box with a spray distance of 20 cm, and heated on a hot plate at 50 °C for half an hour. Finally, a thin transparent top AgNW electrode was spray deposited with a spray distance of 20 cm and dried on a hot plate at 50 °C for 30 minutes. Silver pads were applied to make a low resistance contact to the solar cells for probing. The cell area is 3 mm^2^ which is controlled by the spray mask. After many iterations investigating thickness, curing temperature, stability of the functional layers 18 devices were tested with each containing 8 pixels (cells). Almost all devices functioned although some cells were short circuited at the edges of the top and bottom electrodes.

### Characterizations

The crystallinity of the TiO_2_ compact layer was checked by XRD (Rigaku SmartLab). The morphology and thickness of the TiO_2_ mesoporous layer was observed in a scanning electron microscope (JEOL JSM 7500 FESEM). The surface roughness of the glass fibre textile was measured using surface profilometry (2-D Tencor P-11). The surface roughness of the TiO_2_ compact layer on glass was examined by atomic force microscope (AFM) using a Veeco Innova 3100 instrument. All the devices are measured under dark and illuminated condition by using a digital source meter (Keithley, Model 2400), and a solar simulator (ABET Sun 300) to provide illumination of AM 1.5 conditions. Transmittance measurements were examined using Bentham PV instrumentation (PVE300). Electrochemical impedance spectroscopy (EIS) measurements were performed on AUTOLAB (PGSTAT128n) to characterise electrochemical performance the cells.
